# How probiotics, prebiotics, synbiotics, and postbiotics prevent dental caries: an oral microbiota perspective

**DOI:** 10.1038/s41522-024-00488-7

**Published:** 2024-02-24

**Authors:** Si-Chen Luo, Si-Min Wei, Xin-Tao Luo, Qiong-Qiong Yang, Ka-Hing Wong, Peter C. K. Cheung, Bo-Bo Zhang

**Affiliations:** 1 Guangdong Provincial Key Laboratory of Marine Biology, Department of Biology, College of Science, Shantou University, Shantou, 515063 Guangdong PR China; 2 Research Institute for Future Food, Department of Food Science and Nutrition, The Hong Kong Polytechnic University, Hong Kong, PR China; 3 School of Life Sciences, The Chinese University of Hong Kong, Shatin, New Territories, Hong Kong, PR China

**Keywords:** Applied microbiology, Antimicrobials, Biofilms

## Abstract

Dental caries, a highly prevalent oral disease, impacts a significant portion of the global population. Conventional approaches that indiscriminately eradicate microbes disrupt the natural equilibrium of the oral microbiota. In contrast, biointervention strategies aim to restore this balance by introducing beneficial microorganisms or inhibiting cariogenic ones. Over the past three decades, microbial preparations have garnered considerable attention in dental research for the prevention and treatment of dental caries. However, unlike related pathologies in the gastrointestinal, vaginal, and respiratory tracts, dental caries occurs on hard tissues such as tooth enamel and is closely associated with localized acid overproduction facilitated by cariogenic biofilms. Therefore, it is insufficient to rely solely on previous mechanisms to delineate the role of microbial preparations in the oral cavity. A more comprehensive perspective should involve considering the concepts of cariogenic biofilms. This review elucidates the latest research progress, mechanisms of action, challenges, and future research directions regarding probiotics, prebiotics, synbiotics, and postbiotics for the prevention and treatment of dental caries, taking into account the unique pathogenic mechanisms of dental caries. With an enhanced understanding of oral microbiota, personalized microbial therapy will emerge as a critical future research trend.

## Introduction

Dental caries represents a substantial and pressing global public health challenge, affecting a staggering number of individuals worldwide. Specifically, there are an estimated 64.6 million cases of permanent dentition and an additional 62.9 million cases of primary dentition^1^. *Streptococcus mutans*, one of the major causative bacteria of dental caries that expresses collagen-binding protein, can effectively invade human umbilical vein endothelial cells^2^, thereby leading to the potential development of infective endocarditis.

As dental caries is typically mediated by biofilm, interventions targeting biofilm have become a major strategy for prevention. Adjusting the intake of fermentable substrates in the diet, especially sucrose is an effective approach^3^. The modern dietary environment is characterized by the widespread availability of highly processed and sugary foods, creating a significant challenge in completely abstaining from cariogenic foods. Other interventions include physical clearance (e.g., brushing or using interdental cleaning tools), chemical inhibition (e.g., using chlorhexidine or povidone-iodine), and biological interventions (e.g., using probiotics)^4^. To effectively prevent dental caries, current strategies should aim to suppress the overgrowth of specific cariogenic bacteria by targeting their virulence factors, while also promoting a diverse and healthy resident microbiota^5^. Among these interventions, microbial preparations such as probiotics, prebiotics, synbiotics, and postbiotics have gained significant attention as they offer a more targeted and friendly approach than physical clearance and chemical inhibition.

Meurman and colleagues^6^ were pioneers in introducing probiotics into the field of dentistry. Over time, microbial preparations have gained attention as potential adjunctive therapies for preventing and treating dental caries. These preparations have demonstrated significant effectiveness in inhibiting the growth and biofilm formation of cariogenic bacteria.

This article first provides an overview of the background and pathogenic mechanisms of dental caries, focusing on the virulence factors of cariogenic bacteria *S. mutans*. It then summarizes the latest research progress, mechanism of action, application status, and challenges associated with the use of probiotics, prebiotics, synbiotics, and postbiotics in the prevention of dental caries. Lastly, this article proposes future directions for the development of this field to provide more scientific, standardized, and effective guidelines for the prevention of dental caries from an academic perspective.

## Dental caries

### Background

The oral cavity is a complex ecosystem characterized by various warm, moist microenvironments that provide ideal conditions for microbial growth^7^. A recent study analyzing the oral microbiome identified a total of 1591 microbial species, including bacteria, fungi, archaea, viruses, and protozoa^8^, second only in complexity to the colon^9^. The core oral microbiota in healthy individuals remains relatively stable over seven years^10^, while an imbalanced oral microbiota can lead to dental caries and other oral diseases^11^. Moreover, the diversity of the oral microbial community in severe dental caries is considerably lower than that in healthy individuals^12^. Dental caries arises from an imbalance in the oral microbiota resulting from a complex interplay between the host, diet, and microorganisms^13^.

Of these factors, fermentable carbohydrates, which are commonly found in sweetened foodstuffs, have been identified as particularly important dietary contributors to dental caries^14^. Consumption of sweetened foodstuffs can rapidly increase the concentration of carbohydrates in the oral cavity, leading to a sharp decline in the pH values of biofilm to 4 or even lower^15^. Research has revealed a precise correlation between the areas of acute demineralization on the enamel surface and the highly acidic pH zones created by biofilms^16^. This is attributed to the frequent local pH decreases can disrupt the balance between tooth mineralization and demineralization in the closed microenvironment of the biofilm^17^. Consequently, this leads to mineral loss in teeth, resulting in white spots, cavitation, pulp infections, and even tooth loss^17^.

Differences in oral microflora have been observed between individuals with healthy teeth and those with dental caries. For example, the findings of a study on the oral microbiome of children indicate that the genera *Rothia*, *Neisseria*, and *Haemophilus*, which are among the first colonizers of the oral cavity following birth^18^, are associated with dental health^19^. In contrast, *Prevotella* spp., *S. mutans*, and *Human herpesvirus 4* (EB virus) are more commonly found in children with dental caries^19^. *Actinomycetota* (35.8%) and *Bacillota* (31.2%) were the most common phyla in deep dentin carious lesions, and *Lactobacillus* was the most abundant genus in only 25% of the carious lesions^20^. There is increasing recognition that dental caries is caused by the imbalanced microbiota in the biofilm, also known as dental plaque, rather than by a single pathogen^21^.

### Microorganisms associated with dental caries

The cariogenic bacteria exhibit varying degrees of contribution to the development of dental caries. For decades, *S. mutans* and *Streptococcus sobrinus* have been widely recognized as the major cariogenic agents^22^. It is noteworthy that *S. sobrinus* exhibited superior acidogenicity and aciduricity compared to *S. mutans*, but showed lesser adaptability to the biofilm environment^23^. The cariogenic bacteria within the oral microbiota do not exist as isolated entities but rather interact and influence each other. To a certain extent, *S. mutans* creates a lactic acid-rich environment in carious lesions that facilitates the proliferation of *Veillonella* species^12^, which have been shown to promote the growth of *S. mutans* in biofilm studies^24^.

Additionally, *Candida* species, as a typical fungal representative of cariogenic microorganisms, have emerged as potent secondary cariogenic agents, isolated from 40% to 60% of adult and pediatric caries^25^. *Candida* is a powerful opportunistic caries yeast that relies on the production of short-chain carboxylic acids and proteinases, as well as its ability to adhere to abiotic surfaces and form biofilm^25^. The most common communication between fungi and bacteria in the oral cavity is the mutual interaction between *Candida albicans* and *S. mutans*. The presence of *C. albicans* promotes the growth of *S. mutans*, eliciting notable changes in gene expression and enhancing carbohydrate metabolism^26^. Notably, compared to the mono-species biofilm comprising solely *S. mutans*, there are 393 differentially expressed genes in *S. mutans* within the dual-species biofilm^26^. The glucosyltransferases (Gtf) secreted by *S. mutans* can bind to *C. albicans* and facilitate the conversion of sucrose into exopolysaccharide (EPS), thereby providing binding sites for *S. mutans*^27^.

One study reported that the core microbiota of early childhood caries (ECC) may include *Veillonella parvula*, *Fusobacterium nucleatum*, *Prevotella denticola*, and *Leptotrichia wadei*^28^. On the one hand, this ECC core microbiota promotes the growth and acidogenicity of *S. mutans*, and promotes biofilm formation, albeit with limited acidogenic capacity^28^. On the other hand, it also promotes enamel demineralization in vitro and increases the cariogenic potential of enamel in vivo^28^. Additionally, according to some metagenomic results, the following species are closely associated with dental caries: *Streptococcus gordonii*, *Leptotrichia buccalis*, *V. parvula*, *Actinomyces gerencseriae*, *Propionibacterium acidifaciens*, *Hallella multisaccharivorax*, and *Parascardovia denticolens*^29,30^.

Of the microorganisms associated with dental caries, *S. mutans* is one of the most extensively studied species in this field. Given that *S. mutans* was initially thought to be a major cause of dental caries^31^, it is not surprising that most prevention strategies target this bacterium specifically^29^.

### *Streptococcus mutans*

**Fig. 1:**
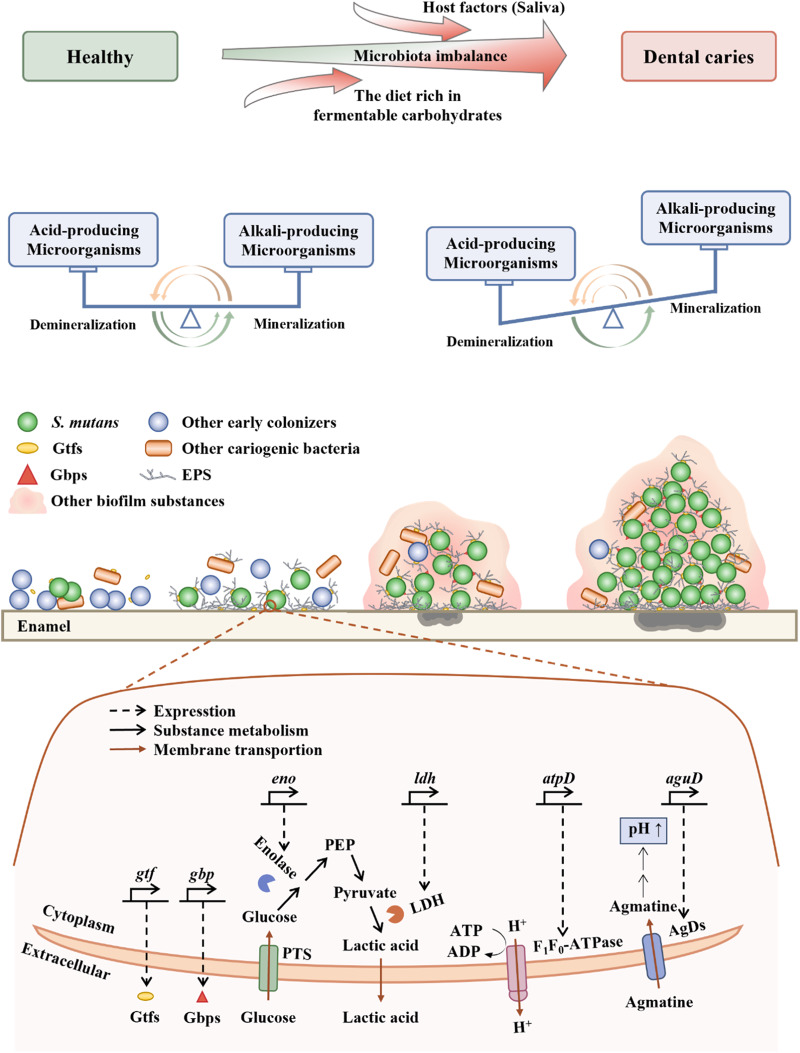
Cariogenic biofilm formation. EPS exopolysaccharides, Gbps Glucan-binding proteins, *gbp* encode Gbps, be related to adhesion, Gtfs Glucosyltransferases, *gtf* encode Gtfs, be related to the synthesis of EPS, *eno* encode Bacteria enolase, be related to glucose uptake, LDH lactate dehydrogenase, *ldh* encode LDH, be related to acid production, PTS phosphotransferase, the glucose uptake system, PEP phosphoenolpyruvate, *atpD* encode F_1_F_0_-ATPase, be related to acid resistance, ATP adenosine triphosphate, ADP adenosine diphosphate, AgDs agmatine deiminase system, *aguD* encode AgDs, be related to acid resistance. Healthy teeth develop dental caries due to the complex interactions between the host, diet, and microorganisms. The microecology of healthy teeth is based on the balance between acidogenic and alkalinogenic microbial activities, as well as the balance between demineralization and remineralization processes. When acidogenic microorganisms become predominant, frequent and high concentrations of acid locally lead to net demineralization of dental enamel, resulting in the formation of cavities. At the micro level, the initial step involves the adhesion of some primitive colonizing microorganisms to the dental enamel. The second step involves the production of EPS by the microorganisms, forming a biofilm. In the third step, acidogenic and acid-tolerant microbial communities, mainly dominated by *S. mutans*, establish a highly acidic microenvironment, leading to demineralization of the dental enamel. In the fourth step, the highly acidic microenvironment confers a growth advantage to *S. mutans*-dominated microbial populations. Taking the virulence factors of *S. mutans* as an example, the synthesis of EPS is primarily mediated by Gtfs. The adhesion process is mainly facilitated by Gbps. Acid production involves the participation of enolase and LDH. Acid tolerance processes primarily rely on the involvement of F_1_F_0_-ATPase and AguD.

When compared with other original colonizing bacteria, *S. mutans* exhibits more advantageous traits by developing a compact biofilm and its distinctive virulence factors^32^. Furthermore, under the regulated control of the quorum-sensing system, *S. mutans* ultimately becomes one of the major cariogenic bacteria. The biofilm in dental caries of primary teeth is a three-dimensional (3D) spherical structure, with *S. mutans* as the core and other bacteria forming the outer layer^16^. This localized area creates an acidic pH environment, leading to severe enamel demineralization^16^. As dental caries progresses, the diversity of the oral microbiota becomes limited^33^. This microbial imbalance eventually leads to the occurrence and development of dental caries. The close association between *S. mutans* and dental caries has been confirmed. Although *S. mutans* is a natural resident of the human oral cavity^34^, an increase in the levels of *S. mutans* should be of concern as it may indicate a clinical precursor to dental caries^35^. Rats infected with human-derived *S. mutans* develop dental caries^36^, and *S. mutans* may be associated with severe-ECC recurrence^37^. These suggest the crucial role of *S. mutans* in the occurrence and development of dental caries. Therefore, further elucidating the pathogenic mechanisms of *S. mutans* (Fig. 1) is essential for the development of effective strategies for caries prevention and treatment. In the following sections, we will discuss the virulence factors and quorum-sensing system aspect of *S. mutans*.

#### Virulence factors

The virulence factor of *S. mutans* can be categorized into four major groups, comprising EPS synthesis, adhesion, acid production, and acid resistance.

##### The synthesis of EPS

The ability of *S. mutans* to exert its pathogenicity is largely attributed to the production of EPS. EPS, a major component of biofilms^38^, consists of extracellular proteins, extracellular DNA, and lipoteichoic acid^39^. The primary component of EPS is glucan, which is synthesized by Gtf^40^, providing binding sites for microorganisms^38^. EPS contributes to the formation of highly organized chemical and physical barriers within the biofilm matrix, facilitating microbial adherence to non-living surfaces^41^, resisting fluid shear stresses^42^, evading host immune responses^43,44^, tolerating antimicrobial agents^45^, and ultimately establishing and maintaining acidic microenvironment in the oral cavity that favors the development of dental caries-associated biofilm communities^42^. Mature biofilms are difficult to remove mechanically due to the enhanced viscoelasticity conferred by EPS^42^. EPS may achieve immune evasion by mediating complement evasion^43^ and limiting the entry of effector molecules from the innate and adaptive immune systems into the biofilm matrix^44^. Chlorhexidine, a commonly used antimicrobial agent in oral care, has limited penetration into deep biofilm layers due to its positive charge, whereas “the fuel” (sucrose), lacking charge, can easily diffuse, facilitated by the negative charge of EPS^45^. The negatively charged surface of *S. mutans* cells enveloped by EPS accumulates protons, and the sieving effect of the glucan structure also plays a role^46^. On one hand, EPS captures and accumulates protons produced externally or by acidogenic microorganisms, aiding in the retention and accumulation of acid within the biofilm^46^. On the other hand, once protons are recruited to the cell surface, they trigger an acid adaptation response, allowing the microorganisms to preemptively counteract acid damage^46^. Deactivation of one or more *gtf* genes significantly reduces the virulence of *S. mutans*, in rodent caries models^47^. In summary, EPS plays a crucial role in enabling *S. mutans* to exert its cariogenic potential. Therefore, inhibiting EPS synthesis may represent a feasible preventive strategy against dental caries^48^.

##### Adhesion

*S. mutans* employs both sucrose-independent and sucrose-dependent pathways to adhere to teeth^47^. The initial adhesion process is primarily mediated by the sucrose-independent pathway, which is subsequently reinforced by the stimulation of glucan synthesis via the sucrose-dependent pathway, ultimately culminating in the formation of biofilms^47^. Glucan-binding proteins facilitate the binding of glucans synthesized from sucrose through glucose transferases. Of these proteins, GbpA exhibits a strong correlation with cariogenicity^49^. On the one hand, it contributes to the formation of strong biofilm structure and is an important protein determining the structure of biofilm. On the other hand, it plays an essential role in linking glucan molecules and is involved in the bacterial adhesion process to teeth.

##### Acid production

After glucose metabolism, dietary carbohydrates produce energy and organic acids as metabolic by-products^50^. The acid-producing activity of *S. mutans* is not only a critical factor contributing to its pathogenicity but also a crucial characteristic leading to dental caries. Bacteria enolase, an enzyme encoded by the gene *eno*, is a primary component of the phosphotransferase system, which is responsible for glucose uptake^51^. Through the rapid catalytic activity of lactate dehydrogenase (LDH), a protein encoded by the *ldh* gene, *S. mutans* UA159 ferments glucose into organic acids^52^.

##### Acid resistance

*S. mutans* employs some acid-resistant mechanisms to cope with the stress of increasing acid production. F_1_F_0_-ATPase, a proton pump encoded by *atpD*^47^, not only pumps out intracellular protons to maintain intracellular pH but also produces ATP to promote bacterial growth and survival^53^. Inhibition of *atpD* expression in *S. mutans* UA159 resulted in a significant decrease in acid adaptation and an increase in cytoplasmic acidity^51^. Additionally, *S. mutans* produces alkali to neutralize acids, as well as export them out of the cells. The agmatine deiminase system plays a crucial role in producing alkalis to overcome acid stress^54^. Amongst its components, the agmatine-putrescine antiporter (AguD), encoded by the *aguD* gene, is of particular importance as it facilitates the intracellular transport of free agmatine^54^. The accumulation of protons on the surface of bacterial cells enveloped by EPS plays a significant role in the acid resistance of *S. mutans*, as mentioned in the “synthesis of EPS” section^46^.

#### Quorum-sensing (QS) system

The QS system regulates virulence and biofilm formation by releasing, sensing, and interacting with diffusion molecules^55^ based on cell density in the surrounding environment^38^. *S. mutans* utilize this system to communicate with each other as a group rather than as separate individuals. The main mechanism for signal feedback is via the two-component signal transduction systems (TCSTS), which enable bacteria to regulate their gene expression^56^. *S. mutans* contains several types of TCSTS, among which *VicRKX* and *ComCDE* are critical in the regulation of biofilm formation, acid resistance, and acid production in response to environmental signals^57,58^. If these regulatory systems fail to function properly, it may lead to a decrease in the cariogenicity of *S. mutans*.

### Dental caries prevention measures—biological interventions

Although plaque is a natural occurrence in teeth from an evolutionary, biological, and nutritional perspective, an imbalance in the microbiome of the oral pathological biofilm can lead to the development of dental caries^59^. Acid-producing cariogenic bacteria, especially *S. mutans*, damage the hard tooth structures in the presence of fermentable carbohydrates^38^.

In recent years, the field of biological intervention has developed some novel strategies. One approach involves using predators, such as *Bdellovibrio*, *Bacteriovorax*, and *Peredibacter*, to eliminate anaerobic Gram-negative bacteria that are periodontal pathogens^60,61^. Given that beneficial bacteria are mostly Gram-positive^62^. Additionally, biological interventions also include the use of specific inhibitors of *S. mutans* proteins, vaccination, and passive immunization strategies with neutralizing bacteria^25,29^. Although some innovative biological intervention strategies such as those mentioned above have emerged, the use of microbial preparations, such as probiotics, prebiotics, synbiotics, and postbiotics, is a more established and popular approach for preventing dental caries.

## Probiotics

### Background

Probiotics were discovered by scholars as early as 1908^63^, and since then the field of studying the health effects of probiotics on the host has gradually developed. In 2013, The International Scientific Association of Probiotics and Prebiotics (ISAPP) defined probiotics as “live microorganisms that, when administered in adequate amounts, confer a health benefit on the host”^64^. Today, probiotics are commonly used by humans to maintain their overall well-being. Although their effectiveness in promoting gastrointestinal health is well-known, research has also shown that probiotics can be effective in preventing and treating various oral diseases, such as dental caries, oral mucositis, and halitosis^65^.

In dentistry, probiotics were first introduced by Meurman and colleagues^6^, who found that *Lacticaseibacillus rhamnosus* GG ATCC 53103 could colonize the human mouth. With further research, probiotics have been found to have a remarkable ability to prevent dental caries. For instance, one study explored the effect of subjects’ own *Lactobacillus* on *S. mutans*^66^. The study has shown that *Lactobacillus* isolated from the oral cavity of subjects can effectively inhibit the growth of *S. mutans*. The most effective species of *S. mutans* were found to be *Lacticaseibacillus paracasei* and *Lactiplantibacillus plantarum*, which are also the most common isolates. Finally, the use of probiotics in the treatment of oral diseases has been found to restore oral microbial balance and reduce the levels of *S. mutans* in dental plaque and saliva^67^.

With different probiotic strains exhibiting unique characteristics, understanding the specifics of each strain is crucial to when prevention and treatment of dental caries. For instance, *L. rhamnosus* GG is a homofermentative *Lactobacillus* that is not considered to be cariogenic because it cannot ferment sucrose or lactose^68^. *Limosilactobacillus reuteri* is an obligate heterofermentative species^68^ that can produce broad-spectrum antimicrobials with good acid-base stability, such as reuterin^69^ and reutericyclin^70^. In addition to *Lactobacillus* spp., *Bifidobacteria* spp. may also be a potential probiotic for preventing and treating dental caries. Yogurt containing *Bifidobacterium* DN-173010 has been reported to significantly reduce the level of *S. mutans*^71^.

### Mechanisms to prevent dental caries

**Fig. 2:**
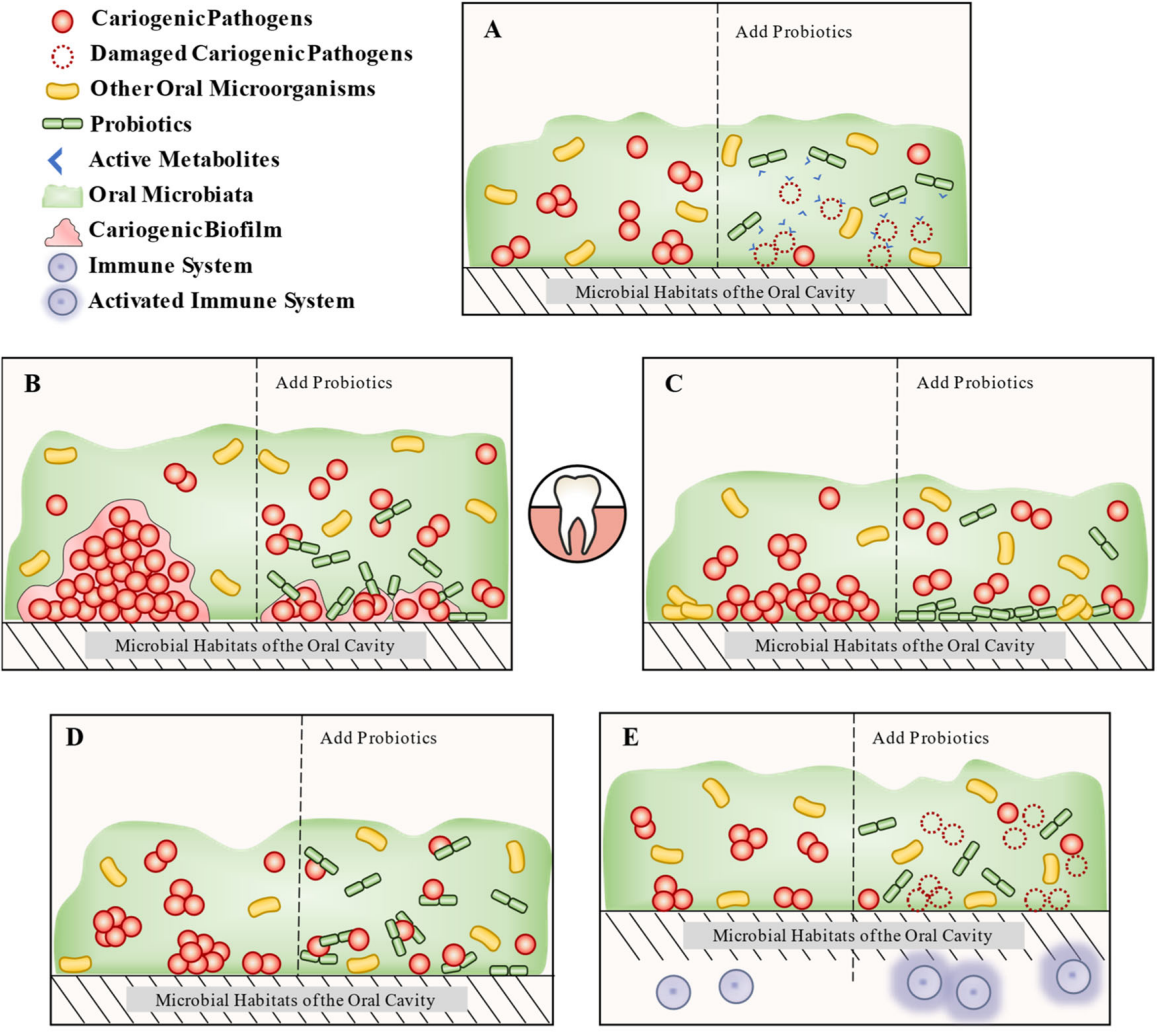
The mechanism of probiotics to prevent dental caries. It is roughly divided into five parts. **A** Production of active metabolites: probiotics directly inhibit cariogenic pathogens by active metabolites (e.g., bacteriocin, enzyme, biosurfactants, organic acids, and hydrogen peroxide), which themselves have bacteriostatic activity. **B** Inhibition of cariogenic microbial biofilm: probiotics can inhibit or remove the biofilm of oral cariogenic microorganisms. **C** Competitive adhesion and colonization: probiotics not only occupy the colonized sites in the oral cavity but also inhibit the adhesion ability of cariogenic microorganisms. **D** Coaggregation with pathogens: probiotics inhibit cariogenic microorganisms colonization in the oral cavity through co-aggregation. **E** Regulation of the immune system: probiotics activate or modulate the host immune system, thereby enhancing the immune response to cariogenic microorganisms (enhances salivary levels of human neutrophil peptides 1–3).

The mechanism through which probiotics can prevent dental caries is similar to that found in the gastrointestinal tract. The principal inhibitory mechanisms include the synthesis of active metabolites, inhibition of cariogenic microbial biofilm, competitive adhesion and colonization, coaggregation with pathogens, and regulation of the immune system (Fig. 2).

#### Production of active metabolites

##### Bacteriocin

Bacteriocin is a cationic antibacterial peptide synthesized by the ribosome^72^ and was first discovered^73^. Bacteriocins can be divided into four different classes, with Classes I and II being the primary focus of most probiotics research^74^. Nisin, a bacteriocin of Type A in Class I, is widely recognized as a small positively charged protein (2–5 kDa) that induces target cells to form membrane pores^72^. Class II bacteriocins kill bacteria by increasing membrane permeability and leaking target bacterial contents^75^. In addition to the above mechanisms, bacteriocins can also inhibit the synthesis of biofilm and cell wall, exert the activities of DNase and RNase, and regulate microbiota^72^.

Protein-protein interaction between the GtfB and LuxS proteins of *S. mutans* and bacteriocin of SD1 in *L. paracasei* was found to reduce the formation of biofilm and the density of microorganisms, as revealed in a simulation study^76^. Following comprehensive bioinformatics analysis and characterization, the bacteriocin in this study was found to be safe for humans. The bacteriocin Mersacidin exerts its bactericidal activity by forming a complex with lipid II, which inhibits cell wall synthesis^77^. The DNase and RNase activities of colicins from E2 to E9 enable them to non-specifically degrade bacterial DNA and RNA^78^. Among these, colicin E2 exhibits potent and long-lasting bactericidal activity, and interestingly, it can specifically target bacteria in complex biological membranes^79^. Bacteriocins are capable of promoting the colonization of producer bacteria in specific niches over a decade, regulating the composition of the microbiota and affecting the host immune system^80^. Both nisin and nisin-producing probiotics can reduce the level of pathogens in biofilm and restore the diversity of strains to a healthy level^81^.

As bacteriocins are polypeptides and proteins, temperature control is crucial to ensure their activity during production and use. *Streptococcus oralis subsp. dentisani* 7746 (AB-Dentisanium^®^), for instance, is optimally concentrated at 30 and 45 °C, with a small reduction in bacteriocin activity at 60 °C^82^. This critical consideration underscores the importance of implementing appropriate temperature regulation strategies in the development and use of bacteriocins for various applications.

##### Enzyme

In addition to bacteriocins, probiotics synthesize a diverse range of enzymes that confer beneficial effects by decomposing biofilms and affecting bacteriocin activity. For instance, *Lactobacillus acidophilus* can secrete lipase to degrade biofilm^83^. Similarly, *Streptococcus salivarius* JH expresses a dextranase enzyme that can hydrolyze the EPS of *S. mutans* and increase the anti-*S. mutans* inhibitory activity of zoocin A, a muralytic bacteriocin^84^. Another example is *Streptococcus* sp. A12, which produces challisin-like proteases that inhibit the production of bacteriocins by *S. mutans*^85^. Additionally, *S. salivarius* M18 produces urease and dextranase to neutralize salivary acidity and reduce plaque formation, respectively^86^.

##### Biosurfactants (BS)

Biosurfactants are amphiphilic substances produced by microbial metabolism that contain both hydrophobic and hydrophilic groups, mainly composed of proteins, sugars, and lipids^87^. The structure of BS can be identified using various techniques such as thin layer chromatography, Fourier Transform Infrared Spectrometer, and Nuclear Magnetic Resonance^88^. Fifty percent of the 40 biosurfactant reports reviewed did not analyze their structure, likely due to the complexity of the structures^87^. Surfactin and/or protein-like biosurfactants (32.5%) are most commonly produced by *Lactobacillus*, with studies on glycoproteins (7.5%)^89^, glycolipid (5%)^90^, and glycolipopeptide (5%)^91^ production being rare.

*Lactobacillus* typically produces surfactin-type biosurfactants, which are protein-rich and can significantly inhibit pathogen adhesion, making them increasingly interesting due to their unique anti-adhesion and anti-biofilm properties^87^. For example, BS produced by *L. reuteri* DSM 17938, *L. acidophilus* DDS-1, *L. rhamnosus* ATCC 53103, and *L. paracasei* B21060 inhibited *S. mutans* and *Streptococcus oralis* adhesion and biofilm formation in a dose-dependent manner on titanium surfaces^92^. In more detail, *L. acidophilus* DSM 20079 produces protein-type BS, which can shorten the chain length of *S. mutans*, interfere with its biofilm formation on glass slides, and down-regulate the gene expression of *gtfB* and *gtfC*^93^. BS produced by *L. rhamnosus* can destroy the physical structure or protein conformation of biofilm, leading to cell lysis^94^. In addition to the appealing antimicrobial activity mentioned above, BS exhibits characteristics of low cytotoxicity and high stability. BS derived from *Lactobacillus* spp. demonstrate comparable low cytotoxicity to rhamnolipids, which are generally regarded as non-toxic products^87^. BS may exhibit better stability compared to other antibacterial substances produced by probiotics. Gudinahe et al.^95^ isolated stable BS from *L. paracasei*. This BS was pH stable within a range of 6–10 and maintained surface activity after incubation at 60 °C for 120 h.

##### Organic acids

Organic acids, such as lactic acid and butyric acid^96^, produced by *Lactobacillus* in the human gastrointestinal tract and other body parts, have widely been recognized as beneficial substances. These organic acids may have a bacteriostatic effect on oral pathogenic microorganisms to a certain degree. For instance, *L. paracasei* Lpc-37 produces the acid that can restrain the growth and biofilm formation of *S. mutans*^97^. However, considering the strong association between dental demineralization and frequent exposure to high concentrations of acid^16^, it raises questions about how the acids produced by probiotics counteract the acids produced by cariogenic bacteria, including *S. mutans*.

These questions may need to be approached from the perspective of the overall caries environment. Cariogenic microorganisms create a highly organized acidic barrier^42^. Prolonged exposure to localized high concentrations of acid leads to localized demineralization rather than average demineralization of the teeth. If the organic acids produced by probiotics can inhibit cariogenic bacteria, including *S. mutans*, and/or their biofilms, they may disrupt this acidic barrier and prevent acid accumulation. Given the presence of its own acid-base microbial balance in the oral cavity^54^, acids that do not accumulate but instead contribute to the acid-base equilibrium in the oral environment appear to pose a lesser threat.

##### Hydrogen peroxide

Certain probiotic species, including *Bifidobacterium bifidum*, *Lactobacillus johnsonii*, *Lactobacillus crispatus*, and *Lactobacillus jensenii*, produce hydrogen peroxide to exert antibacterial effects^98^. Hydrogen peroxide acts on the pathogenic bacteria’s epithelium, leading to their death^98^. Moreover, hydrogen peroxide has the potential to regulate species composition within the oral cavity^99^. Notably, cariogenic species such as *S. mutans* are highly susceptible to hydrogen peroxide toxicity^100^. However, the antibacterial activity of *L. paracasei* cell-free supernatant (CFS) was significantly reduced after catalase treatment, indicating the involvement of hydrogen peroxide in its bacteriostatic effects^101^. It should be noted that hydrogen peroxide may not retain its bacteriostatic function after processes such as freeze-drying, owing to the ease of its decomposition^102^.

#### Inhibition of cariogenic microbial biofilm

Dental caries is commonly mediated by biofilm. A crucial property of probiotics is the ability to inhibit or eliminate the growth of biofilms and pathogenic microorganisms in the oral cavity. Some strains have often been reported for their anti-biofilm activity, including *S. oralis* 89a, *Limosilactobacillus fermentum* TCUESC01, *L. acidophilus* 4A, and *Bifidobacterium longum subsp. longum*^103^. For instance, *Lacticaseibacillus casei* ATCC 393, *L. reuteri* ATCC 23272, *L. plantarum* ATCC 14917, and *Ligilactobacillus salivarius* ATCC 11741 may suppress the biofilms of *S. mutans* by down-regulating genes such as *gtfB*, *gtfC*, and *gtfD* in *S. mutans*^104^. Interestingly, probiotics are capable of inhibiting fungi from transitioning into pathogenic forms. It has been demonstrated that *L. rhamnosus* LR32, *L. casei* L324m, and *L. acidophilus* NCFM exhibit the ability to impede the initial stages of hyphal formation, which is a crucial step in the pathogenesis of *C. albicans*^105^. A probiotic combination consisting of *Lactobacillus helveticus* CBS N116411, *L. plantarum* SD5870, and *S. salivarius* DSM 14685 significantly down-regulated the expression of genes involved in yeast-hypha transition in *C. albicans*, including *EFG1* (hyphae-specific gene activator), *SAP5* (secreted protease), *ALS3* (adhesin/invasin) and *HWP1* (hyphal wall protein)^106^. In vitro, biofilm models are continuously improving to replicate more closely the conditions found within the human body. Based on the specific research objectives, researchers can opt for models of interest, such as an experimental abutment mimicking the macro- and microstructure of a dental implant^107^.

The combination of *L. rhamnosus* and collagen peptides was found to significantly increase the pH of the medium in the early stages of biofilm formation^108^. The qPCR results showed that this combination down-regulated several crucial genes linked to acid production and acid tolerance, including *eno*, *ldh*, and *atpD*. Moreover, probiotics may also exert antibacterial effects by interfering with QS. A study revealed that *comD*, *vicR*, and *vicK* genes were down-regulated in planktonic and biofilm forms of *S. mutans* when exposed to CFS with *Lactobacillus*^104^. This effect may explain the reduced adherence and biofilm formation of *S. mutans* observed in scanning electron microscopy experiments.

#### Competitive adhesion and colonization

One of the key characteristics of probiotics contributing to their health effects is their capacity for outcompeting oral pathogens concerning adhesion and colonization^109^. For example, *L. reuteri* LR6 displayed the most substantial adhesion capabilities among eight tested probiotic strains, which corresponded to a higher ability for inhibiting the adherence of pathogens to Caco-2 cells^110^. Enhanced colonization efficacy by *S. salivarius* M18 resulted in stronger anti-caries activity as evidenced by a reduction in plaque scores and *S. mutans* levels^111^. *Levilactobacillus brevis* KCCM 202399 inhibited the adherence of *S. mutans* KCTC 5458 by reducing the self-aggregation, cell surface hydrophobicity, and EPS production of *S. mutans*^112^.

Interestingly, probiotics can reduce pathogen adhesion even without direct contact. Saliva treated with probiotics was shown to reduce the adhesion of *S. mutans* to hydroxyapatite surfaces (a model for enamel)^113^. Further studies showed that the above salivary membrane lacked two proteins: salivary lectin gp340, the primary receptor for *S. mutans* in the salivary membrane, and salivary peroxidase, an innate defense factor found in human saliva^114^.

#### Coaggregation with pathogens

Coaggregation is among the advantageous properties of probiotics as it allows them to form a barrier that impedes pathogen colonization^115^. In a study, six out of 624 lactic acid bacteria were found to exhibit specific coaggregation with *S. mutans* in vitro^116^. These species were identified as *L. paracasei* and *L. rhamnosus*. It was discovered that this coaggregation mechanism is highly resilient to both hyperthermia and protease, and does not rely on lectins, nor is it impacted by saliva.

#### Regulation of the immune system

In addition to their direct effects on pathogenic microorganisms or biofilm, probiotics are known to activate and modulate the host’s immune system^117^. Clinical studies have shown that daily or tri-weekly consumption of *L. paracasei* SD1 in patients with severe ECC significantly enhances salivary levels of human neutrophil peptides 1–3 with a broad bactericidal activity and reduces *S. mutans* levels, potentially slowing the progression of caries^118^. Furthermore, the consumption of milk containing *L. paracasei* SD1 for six months increased salivary immunoglobulin A levels, and this increase is positively correlated with a load of *L. paracasei*^119^.

Certain strains of *Streptococcus thermophilus*, such as ST1342, ST1275, and ST285 activate the innate immune response and stimulate the secretion of interleukin-1β, tumor necrosis factor-α, interleukin-6, and interferon-γ by monocytes, thereby contributing to the elimination of pathogens^103^. Commercial *L. paracasei* DG has immunostimulatory activity by boosting tumor necrosis factor-α, interleukin-6, and Chemokine (C-C motif) ligand 20 expressions in human monocyte leukemia cell^120^. These findings suggest that probiotics can enhance the host’s immune response against pathogenic microorganisms, providing a potential approach to preventing and treating infectious diseases.

### The application vehicle

The colonization of probiotics in the oral cavity may be influenced by the choice of the delivery vehicle^68^. A range of vehicles is available for delivering probiotics, including dairy products, ice cream, cereal, pacifiers, chewing gum, curd, juice, and mouth wash (Table 1). Table 1 also mentions the test species, dose, and efficacies.

**Table 1:** Application vehicle and therapeutic effects of probiotics

Vehicle	Test strain	Dose	Frequency	Sample	Result	Reference
Milk powder	*L. paracasssei*	5 × 10^7^ CFU	once daily for 3 months	124 children aged 1.5–5	reduced the count of *S. mutans* in saliva and delayed the development of new dental caries	187
Milk	*L. paracasei*	7.5 × 10^9^ CFU	once daily for 4 weeks	30 orthodontically treated nonsyndromic cleft lip and palate patients with a mean age of 19	reduced the count of *S. mutans*, while increasing the count of *Lactobacillus* and the colonization	188
Yogurt	*B. animalis*	2 × 10^8^ CFU	once daily for 2 weeks	49 healthy children aged 6–12	could not reduce the levels of salivary *S. mutans* and *Lactobacillus*	189
Yogurt	*B. lactis*	unclear	once daily for 2 weeks	30 individuals aged 10–30 undergoing orthodontic treatment	reduced total microbial counts in dental plaque	190
Yogurt	*B. lactis* BB12	1 × 10^6^ CFU	300 g daily for 2 weeks	66 students aged 18–30 with initial stages of dental caries	reduced the count of *S. mutans* and *Lactobacillus* in the probiotic group	191
Cheese	*L. acidophilus* NCFM or *L. rhamnosus* Lr-32 (DuPont^TM^ Danisco^®^, São Paulo, Brazil)	1 × 10^8-9^ CFU/g each strain	50 g daily for 16 weeks	60 elderly denture wearers	reduced the colonization of oral *Candida*	192
Cheese	*L. rhamnosus* GG and *L. rhamnosus* LC705	1 × 10^7^ CFU/g each strain	5 × 15 g daily for 3 weeks after a meal or snack	74 adults aged 18–35	reduced the count of *S. mutans* during the post-treatment period	193
Cheese	*L. casei* LAFTIL26	1 × 10^6^ CFU/g	50 g twice daily for 2 weeks with breakfast and dinner meals	60 adults with a mean age of 28	could not reduce the count of *S. mutans and Lactobacillus* in the probiotic group	194
Ice cream	*B. lactis* Bb-12 and *L. acidophilus* La-5	1 × 10^6^ CFU each strain	once daily for 7 days	60 healthy children aged 6–12	reduced the count of salivary *S. mutans*	195
Cereal	*L. paracasei* F19	1 × 10^8^ CFU	once daily for 9 months	179 infants aged 4 months	no impact on the frequency of dental caries, mutans streptococci, or lactobacilli	196
Novel slow-release pacifier	*B. animalis lactis* BB-12	1 × 10^10^ CFU	twice daily for 2 years	106 infants aged 1–2 months	no impact on the oral colonization of *B. animalis lactis* BB-12 and mutans streptococci in the early administration	197
Chewing gum	*L. reuteri* ATCC 55730 and ATCC PTA 1	1 × 10^8^ CFU/gum each strain	three times daily after meals for 3 weeks	80 healthy adults aged 21–24	significantly reduced the levels of salivary mutans streptococci	198
Curd	*L. acidophilus* and *B. lactis* BB12 (Mother dairy b-activ Plus®)	unclear	once daily for 7 days before breakfast	60 caries-free adults aged 20–25	Significantly improved salivary pH and reduced the count of salivary *S. mutans*	199
Curd	*L. acidophilus-SD* 5221 (Active Plus; Nestle, Chennai, India)	1 × 10^9^ CFU	with their lunch for 30 days	60 orthodontic patients aged 14–29	significantly reduced the levels of *S. mutans* in the plaque around the brackets	200
Carrot-pineapple juice (Gefilus^®^)	*L. rhamnosus* GG	5 × 10^6^ CFU/mL	five times a week for 7 months	530 healthy children aged 3–6	reduced the count of *S. mutans* and the risk of dental caries	201
Mouthwash (ProBiora^3,TM]^)	*S. oralis* KJ3sm, *S. uberis* KJ2sm, and *S. rattus* JH145	10^6^ or 10^8^ CFU each strain	twice daily for 4 weeks	20 healthy adults aged 21–35	reduced the levels of *S. mutans*	202

*CFU* colony forming units, *B. animalis Bifidobacterium animalis, S. uberis Streptococcus uberis, S. rattus Streptococcus rattus*.

Among these vehicles, dairy products are considered ideal carriers due to their inherent beneficial characteristics^121^. Among the dairy products, liquid substrates, such as milk and yogurt, were found to be more effective in reducing *S. mutans* levels^122^. For individuals who are allergic to dairy products, alternative carriers may be selected, as illustrated in Table 1. The buffering capacity of milk helps to reduce acid production, while its colloidal nature appears to protect enamel^123^. In addition, milk contains calcium and calcium lactate, which may have a preventive effect against caries^124^, and can reduce the colonization of pathogenic microorganisms^125^. Furthermore, milk and cheese promote the dominance of casein phosphopeptides which are known to play a key role in biomineralization^126^. A systematic review and meta-analysis showed that dairy products containing probiotics had a significant impact in reducing *S. mutans* and raising salivary pH^122^.

Interestingly, the slow release of probiotics can also be achieved using appropriate embedding materials. For instance, *L. paracasei* 28.4-gellan formulations were recently found to release the probiotic for over 24 h^127^. *L. paracasei* in this state was able to inhibit *S. mutans* in both the floating and biofilm states, significantly reduce the generation of EPS, and downregulate the *luxS*, *brpA*, *gbpB*, and *gtfB* genes.

### Controversy

Some researchers have taken a critical view of the idea that probiotics can prevent dental caries, primarily focusing on its safety and potential cariogenic effects. The majority of probiotics are not derived from the oral microbiota but from fecal samples, and some even come from animals^82^. Therefore, it is necessary to thoroughly evaluate their safety before clinical use. Probiotics may pose a risk to individuals with damaged barriers or low immunity, such as bacteremia^128^ because high concentrations of administration are key to medication. Regarding the cariogenic issue, after conducting a meta-analysis of 50 experiments related to dental caries and periodontal diseases, Gruner and colleagues concluded that there is insufficient evidence to support the use of probiotics in treating dental caries^129^. Subjects with active dental caries showed higher levels of *S. mutans*, *Actinomyces* sp. strain B19SC, and *Lactobacillus* spp. as detected by PCR-based methods^130^. The major lactic acid bacteria identified from carious lesions, including both adults and children, include *L. fermentum*, *L. casei/paracasei*, *L. salivarius*, *L. rhamnosus*, *L. plantarum*, and *L. gasseri*^131^. *Bifidobacterium dentium*, considered a late marker for dental caries progression, was not found in the oral cavity of caries-free individuals but was detected in 30.8% of caries cases in a study of 56 participants^132^.

It is unreasonable to conclude a causal relationship between lactic acid bacteria and caries if there is a strong correlation between lactic acid bacteria and caries scores^133^. Lactic acid bacteria have a relatively low affinity for teeth, and their ability to form biofilms in vitro is much weaker than that of *S. mutans*^131^. The attachment and proliferation of secondary invaders including *Bifidobacterium* and lactic acid bacteria requires initiation of caries by major caries promoters including *S. mutans* to create an anaerobic acidic environment rich in carbohydrates^132,133^. The destruction of dentin is not enough by lactic acid alone but also requires proteolytic activity, because the main component of dentin^134^ is more than the extracellular matrix dominated by type I collagen. However, lactic acid bacteria, including *L. rhamnosus*, *L. casei/paracasei, L. salivarius*, *Lactobacillus vaginalis*, *Lactobacillus gasseri*, *Limosilactobacillus oris*, and *L. fermentum*, have a greater propensity to bind to collagen rather than degrade it based on genomic analysis^133^.

In conclusion, the effectiveness of probiotics in preventing dental caries remains controversial. It is beneficial to prevent dental caries by exploring the roles of each microflora in the transition from oral health microbiota to cariogenic microbiota. In particular, from the perspective of oral microecology, the diet composition, host immune environment, and the physical and chemical characteristics of the oral cavity, especially the teeth, should be fully considered. Therefore, in recent years, researchers have become increasingly interested in exploring the benefits of prebiotics, synbiotics, and postbiotics in preventing dental caries, especially in terms of advantages over probiotics.

## Prebiotics

In 1995, prebiotics were defined as “non-digestible food ingredient that beneficially affects the host by selectively stimulating the growth and/or activity of one or a limited number of bacteria already resident in the colon”^135^. However, with advancements in scientific research, ISAPP deemed the definition of prebiotics as “a substrate that is selectively utilized by host microorganisms conferring a health benefit” more appropriate in 2017^136^. Prebiotics present a safe and effective alternative to probiotic intervention since they are not live bacteria and are less susceptible to environmental factors affecting probiotic survival and efficacy. The following section will discuss prebiotics according to their different types, including their mechanisms of action, efficacies, and other related aspects.

### Sugar

Interestingly, certain sugars exhibit prebiotic properties. D-tagatose, a non-cariogenic sugar, is a potential prebiotic that offers lower calories and a lower glycemic index than sucrose^137^. Notably, the saliva of individuals with good oral health is rich in D-tagatose^138^. D-tagatose may inhibit the growth of *S. mutans* and *S. gordonii* by affecting glycolysis and its downstream metabolism, but it does not affect *S. oralis*^138^. Encouragingly, chewing gum containing D-tagatose has been shown to inhibit the growth of *S. mutans*^139^.

In addition to D-tagatose, other sugars such as xylose and arabinose are considered potential prebiotics, with the capacity to not only inhibit the growth of *S. mutans* but also promote the growth of *Lactobacillus*^140^. This dual action is particularly advantageous, as it may help restore the balance of the oral microbiome. Given the promising prebiotic properties of these sugars, further research is needed to assess their effectiveness in vivo and their potential side effects.

### Sugar alcohol

Sugar alcohols, such as xylitol, sorbitol, maltitol, and erythritol, have been shown to exhibit prebiotic properties that can enhance oral health. Xylitol, a five-carbon polyol sweetener^141^, is considered an oral-specific prebiotic according to the new definition established in 2017^142,143^. It offers numerous benefits, including the enhancement of remineralization, decrease of the pH of dental plaque, reduction of the level of *S. mutans* in saliva, reduction of the insoluble dextran in the biofilm of *S. mutans*, and reduction of dental caries incidence^144–146^. However, xylitol loses its effect in the presence of fructose or sucrose^147^. Other sugar alcohols, such as sorbitol, maltitol, and erythritol, have also been shown to inhibit dental caries^148,149^.

### Oligosaccharides

In addition to sugar alcohols, oligosaccharides are also being investigated as prebiotics. Human milk oligosaccharides (HMOs), the third most abundant ingredient in human milk, are often added to infant formula^150^. Galacto-oligosaccharides (GOS) and 2′-fucosyllactose, the most abundant HMOs, were found to reduce the EPS-mediated adhesion of *S. mutans* DSM 20523 to the glass surface, indicating their potential as prebiotics for oral health promotion^150^. In addition, GOS, glucomannan hydrolysates, and mannose have also been shown to inhibit pathogen adhesion to epithelial cells by binding to the pathogen’s lectins/pili^151^. However, it should be noted that non-digestible and/or non-absorbable sugar alcohols and oligosaccharides, though beneficial for health, excessive intake may lead to significant diarrhea^152^. Therefore, further research is needed to determine the optimal dosage and duration of intake to minimize such adverse effects.

### Arginine

Arginine is a widely studied oral prebiotic that has been shown to exhibit various beneficial effects on oral health^38^. These benefits include the promotion of alkaline substance production, the mitigation of tooth demineralization, and the suppression of biofilm formation. Specifically, arginine can inhibit the growth of *Candida*^153^ and reduce enamel demineralization^154^. Furthermore, L-arginine has been found to enhance the alkali-producing ability of arginine-solubilizing bacteria, such as *Streptococcus sanguinis* and *S. gordonii*^155^, thereby making the biofilm environment unsuitable for cariogenic microflora by increasing the pH. Interestingly, L-arginine was found to significantly reduce the amount of insoluble EPS by 3-fold, targeting *gtfB*^156^.

Numerous studies have used toothpaste containing arginine to explore the mechanism underlying its prebiotic effects on oral health in depth. An in vivo study conducted on the oral ecosystem has revealed that the presence of arginine in toothpaste enhances the arginolytic capacity of human saliva while reducing its sucrose metabolic activity^157^. Additionally, it promotes a shift in the composition of salivary microbiota towards a healthier ecological state. Notably, tubes of toothpaste containing arginine and fluoride were more effective at preventing and reversing early caries lesions^158^ and significantly increased re-mineralization compared with fluoride-only toothpaste^159^. Using toothpaste containing fluoride and arginine was also associated with an increase in gene expression associated with the arginine deiminase pathway, according to metagenomic and metagenomic data^160^. Moreover, the use of toothpaste containing fluoride and arginine was found to reduce caries bacteria and promote healthier microbial communities.

Interestingly, arginine may stimulate the production of effective substances by probiotics. Exogenous arginine has been found to increase the expression of the *S. gordonii spxB* gene, which encodes a pyruvate oxidase (SpxB), thereby promoting hydrogen peroxide production^156^. Additionally, using Mg^2+^ as a cofactor of the catalytic activity of SpxB^161^ has been shown to increase the production of H_2_O_2_ and promote the abundance of SpxB in *S. sanguinis* and *S. gordonii*^161^.

### Urea and nitrates

Urea and nitrates have been investigated as potential oral prebiotics. Urea is considered a prebiotic^162^ due to its ability to be converted into ammonia or ammonium and bicarbonate ion by bacteria possessing urease, such as *S. salivarius*, *Actinomyces naeslundii*, and *Haemophilus* spp., thereby neutralizing acids in the oral cavity^163^. Nitrate-reducing bacteria in the oral cavity convert salivary nitrate to nitrite, which is subsequently reduced to nitric oxide^164^. All three compounds have been shown to restrict the growth of pathogenic bacteria^165,166^. Nitrate has demonstrated the ability to reduce caries incidence^164,165^ and inhibit bacteria commonly associated with dental caries, such as *S. mutans* NCTC 10499, *L. casei*, and *A. naeslundii*, as well as periodontal disease-related bacteria including *F. nucleatum*, *Eikenella corrodens*, and *Porphyromonas gingivalis*^167^. However, it can lead to an increase in the levels of *Neisseria* and *Rothia*, genera associated with oral health and nitrate reduction^168^. Salivary nitrate supports nitrate respiration by anaerobic microorganisms, ultimately increasing oral pH through various mechanisms. These mechanisms include competition for carbon sources with acid-producing fermentation processes, generation of hydroxyl ions, and dissimilation of nitrate into ammonium to consume organic acids^168,169^. In addition to its impact on dental caries, dietary nitrate not only benefits oral health by significantly reducing gum inflammation^170^ but also contributes to overall health by lowering systemic blood pressure^171,172^, promoting vascular health^172^, and even potentially improving vascular function in patients with hypercholesterolemia^173^. Considering the systemic health effects of nitrate, further investigation is warranted to explore other mechanisms through which nitrate can prevent dental caries.

Both probiotics and prebiotics have beneficial effects on health and combining them appropriately for co-administration represents another approach to drug administration. The following section will further elaborate on this combination.

## Synbiotics

The combined use of probiotics and prebiotics has demonstrated superior therapeutic effects compared to their utilization^174^. The term “synbiotics” was first coined in 1995 by Gibson et al. to describe the combination of probiotics and prebiotics^135^. ISAPP updated the definition of synbiotics in 2020, stating that it is a mixture comprising live microorganisms and substrate(s) selectively utilized by host microorganisms that confers a health benefit on the host^175^. It is not surprising that synbiotics have received less attention compared to probiotics, as it was proposed relatively later.

Several studies have indicated potential oral health benefits associated with synbiotic use. For instance, Nunpan et al.^176^ demonstrated that the synbiotic composed of *L. acidophilus* in combination with GOS and fructo-oligosaccharides can significantly inhibit the growth of *S. mutans*. Tester and Al-Ghazzewi^177^ found that synbiotics composed of Konjac glucomannan hydrolysates and *L. acidophilus* reduced the levels of *S. mutans* in vitro. In another study, Kojima et al. proposed a novel symbiotic^140^. They screened five strains of lactobacilli using sugar assimilation tests with 12 different saccharides, among which the three most promising prebiotics were found to be arabinose, xylose, and xylitol. The selected lactobacilli significantly inhibited the production of water-insoluble glucan by *S. mutans*^140^.

Considering the significant role of excessive acid in dental caries development, the selection of synbiotics comprising prebiotics capable of maintaining a high pH oral environment represents an innovative and intelligent approach. Synbiotics composed of 2% L-arginine and *L. rhamnosus* not only reduced the biomass of *S. mutans* biofilm but also decreased lactate content in spent media, resulting in no significant decline in pH within 24 h^178^. This suggests that synbiotics modulate the ecology of dental plaque. Remarkably, this study also observed that the addition of L-arginine promoted the positive utilization of amino acid biosynthetic pathways by *L. rhamnosus*, thus facilitating its proliferation. These findings indicate that the selection of synbiotics capable of pH regulation may offer stronger advantages in the oral cavity.

These findings provide promising evidence for the development of synbiotics as a novel approach to improving oral health. Further research is needed to establish the safety and efficacy of different synbiotic combinations. It is hoped that these advances will lead to the development of innovative yet effective strategies for promoting oral health, thereby improving overall health outcomes.

## Postbiotics

In 2021, ISAPP defined “postbiotics” as “the preparation of inanimate microorganisms and/or their components that confer a health benefit on the host”^179^, excluding essentially purified metabolites such as butyric acid^179^. Before this, the term and definition of postbiotics were not officially standardized and unified. Postbiotics have also been referred to as “paraprobiotics”, “heat-killed probiotics”, “ghost probiotics”, “non-viable probiotics” and “bacterial lysates”^180^. Over the past few years, the research direction of postbiotics has garnered increasing attention from researchers and has gradually become a hot research topic. Postbiotics are considered superior to probiotics due to their good acid–base and thermal stability, ease of storage and use, and high safety, as many probiotics are sensitive to oxygen and heat^179,181^. The feature of postbiotics enables them to be added to common products such as toothpaste, chewing gum, natto, potato chips, popcorn, and suckable candies^179,182^. Another advantage of postbiotics is that microorganisms cannot be isolated from commercial products, thus enabling product developers to maintain ownership of their components^179^.

The main methods of preparing postbiotics are heat inactivation of bacterial cells and preparation of CFS. In addition, other inactivated technologies, such as electric field, ultrasonication, high pressure, X-rays, high voltage electrical discharge, magnetic field heating, moderate magnetic field, and plasma technology, are also available^179,183^. It should be noted that the mode of inactivation may affect the activity of postbiotics to some extent. For example, in one study, the activity of CFS of *S. oralis subsp. dentisani* 7746 concentrated at 60 °C was lost, while CFS concentrated at 30 °C or 45 °C retained its activity^82^. The ability of heat-killed *L. reuteri* to adhere to and inhibit pathogens was significantly reduced compared to *L. reuteri*^110^, possibly due to the alteration of physical and chemical properties caused by heat treatment^184^.

Although postbiotics do not contain live microorganisms, this does not imply that inactivated bacteria have completely lost all beneficial properties. For example, heat-killed *Bifidobacterium animalis* BB12 still can reduce the cariogenicity of biofilm in vitro^185^, indicating that heat-killed bacteria did not completely lose all their beneficial properties. As another example, *L. paracasei* DSMZ16671 maintained its ability to co-aggregate with *S. mutans* after heat-killed treatment (autoclaved at 121 °C for 20 min)^116^. There was even a study that showed that *L. rhamnosus* CNCM-I-3698 and *Companilactobacillus farciminis* CNCM-I-3699 had a greater ability to exclude pathogens and adhesion after heat-killed treatment^186^. The inactivation process may lead to the disruption of bacterial cell structures, making bioactive molecules more exposed and accessible for utilization^179^. Postbiotics, which are mixtures of various components, may exert their health effects on the oral cavity through multiple mechanisms. These mechanisms can be independent or cooperative and may resemble the previously described mechanisms of probiotic health effects. In the interest of brevity, we will not reiterate these mechanisms here.

In the context of dental caries prevention and treatment, the types of postbiotics commonly utilized include CFS and heat-killed probiotics. However, it is worth mentioning that research in fields beyond the oral cavity has explored a broader range of postbiotic types, such as peptidoglycans, lipopolysaccharides, and pili^179^. Additional investigations into the mechanisms by which other types of postbiotics act concerning dental caries have the potential to provide innovative approaches for the prevention and treatment of this condition. Furthermore, further studies are warranted to assess the safety and effectiveness of postbiotics, thereby contributing to the improvement of overall health outcomes.

## Conclusion and future perspectives

Dental caries prevention and treatment is a critical issue in oral healthcare. The traditional prevention and treatment methods have mainly focused on physical removal and chemical inhibition. While minimally invasive dental restoration can treat cavities, it fails to address the underlying causes of new caries formation. Thus, safer, more effective, and personalized preventive and treatment strategies are urgently needed. Extensive research has been conducted on the application of microbial preparations, such as probiotics, prebiotics, synbiotics, and postbiotics, in the prevention and treatment of dental caries, with promising outcomes. These microbial preparations can modulate the balance of oral microbiota by introducing beneficial microorganisms or inhibiting pathogenic ones. This review aims to help overcome theoretical obstacles to the successful clinical application of microbial preparations in preventing and treating dental caries.

In conclusion, investigating the potential applications of microbial preparations in the prevention and treatment of dental caries is an essential research avenue in the field of oral microbiology. The successful application of microbial preparations in the clinical setting can provide crucial support and greater assurance for oral health. Dental caries pathogenesis is closely associated with the balance of oral microbiota. Therefore, future research should focus on obtaining a deeper understanding of the characteristics and interrelationships among various beneficial and harmful microbial populations. With such comprehensive research, it is advantageous to develop more effective microbial preparations and optimal allocation methods. Developing personalized treatment plans is the current trend, and by devising microbial preparations that are effective for different dental caries risk and medication risk populations, personalized prevention and treatment of dental caries can be achieved. Furthermore, personalized treatment schemes are not limited to single microbial preparations and can be used alongside other treatment modalities to enhance the overall therapeutic outcomes and achieve the goal of curing dental caries. Improving efficacy and safety is a critical direction for future research. Based on a further exploration of the mechanisms of action of microbial preparations, refining and optimizing formulations according to research findings can enhance the therapeutic effects and safety of microbial preparations.

## References

[1] Wen PYF, Chen MX, Zhong YJ, Dong QQ, Wong HM (2022). Global burden and inequality of dental caries, 1990 to 2019. J. Dent. Res..

[2] Nomura R (2020). Potential involvement of *Streptococcus mutans* possessing collagen binding protein Cnm in infective endocarditis. Sci. Rep..

[3] Philip N, Suneja B, Walsh LJ (2018). Ecological approaches to dental caries prevention: paradigm shift or shibboleth?. Caries Res..

[4] Yu OY, Lam WY, Wong AW, Duangthip D, Chu CH (2021). Nonrestorative management of dental caries. Dent. J..

[5] Marsh PD, Head DA, Devine DA (2015). Ecological approaches to oral biofilms: control without killing. Caries Res..

[6] Meurman JH, Antila H, Salminen S (1994). Recovery of *Lactobacillus* strain GG (ATCC 53103) from saliva of healthy volunteers after consumption of yoghurt prepared with the bacterium. Microb. Ecol. Health Dis..

[7] Chattopadhyay I (2022). Can metagenomics unravel the impact of oral bacteriome in human diseases?. Biotechnol. Genet. Eng. Rev..

[8] Achtman M, Zhou Z (2020). Metagenomics of the modern and historical human oral microbiome with phylogenetic studies on *Streptococcus mutans* and *Streptococcus sobrinus*. Philos. Trans. R. Soc. B Biol. Sci..

[9] Wade WG (2013). The oral microbiome in health and disease. Pharmacol. Res..

[10] Rosier BT, Marsh PD, Mira A (2018). Resilience of the oral microbiota in health: mechanisms that prevent dysbiosis. J. Dent. Res..

[11] Kilian M (2018). The oral microbiome—friend or foe?. Eur. J. Oral. Sci..

[12] Kanasi E (2010). Clonal analysis of the microbiota of severe early childhood caries. Caries Res..

[13] Hajishengallis E, Parsaei Y, Klein MI, Koo H (2017). Advances in the microbial etiology and pathogenesis of early childhood caries. Mol. Oral Microbiol..

[14] Forssten SD, Bjorklund M, Ouwehand AC (2010). *Streptococcus mutans*, caries and simulation models. Nutrients.

[15] Gong Y (2009). Global transcriptional analysis of acid-inducible genes in *Streptococcus mutans*: multiple two-component systems involved in acid adaptation. Microbiology.

[16] Kim D (2020). Spatial mapping of polymicrobial communities reveals a precise biogeography associated with human dental caries. Proc. Natl. Acad. Sci. USA.

[17] Peres MA (2019). Oral diseases: a global public health challenge. Lancet.

[18] Palmer RJ (2017). Interbacterial adhesion networks within early oral biofilms of single human hosts. Appl. Environ. Microbiol..

[19] Baker JL (2021). Deep metagenomics examines the oral microbiome during dental caries, revealing novel taxa and co-occurrences with host molecules. Genome Res..

[20] Liu G, Wu C, Abrams WR, Li Y (2020). Structural and functional characteristics of the microbiome in deep-dentin caries. J. Dent. Res..

[21] Jenkinson HF, Lamont RJ (2005). Oral microbial communities in sickness and in health. Trends Microbiol..

[22] Kazemtabrizi A, Haddadi A, Shavandi M, Harzandi N (2020). Metagenomic investigation of bacteria associated with dental lesions: a cross-sectional study. Med. Oral Patol. Oral Cir. Bucal.

[23] Peterson SN, Snesrud E, Schork NJ, Bretz WA (2011). Dental caries pathogenicity: a genomic and metagenomic perspective. Int. Dent. J..

[24] Kluytmans J, van Belkum A, Verbrugh H (1997). Nasal carriage of *Staphylococcus aureus*: epidemiology, underlying mechanisms, and associated risks. Clin. Microbiol. Rev..

[25] Sivamaruthi BS, Kesika P, Chaiyasut C (2020). A review of the role of probiotic supplementation in dental caries. Probiotics Antimicrob. Proteins.

[26] He J (2017). RNA-Seq reveals enhanced sugar metabolism in *Streptococcus mutans* co-cultured with *Candida albicans* within mixed-species biofilms. Front. Microbiol..

[27] Priya A, Selvaraj A, Divya D, Karthik Raja R, Pandian SK (2021). In vitro and in vivo anti-infective potential of thymol against early childhood caries causing dual species *Candida albicans* and *Streptococcus mutans*. Front. Pharmacol..

[28] Chen J (2021). Core microbiota promotes the development of dental caries. Appl. Sci..

[29] Belda-Ferre P (2012). The oral metagenome in health and disease. ISME J..

[30] Pang L (2021). Metagenomic analysis of dental plaque on pit and fissure sites with and without caries among adolescents. Front. Cell. Infect. Microbiol..

[31] Loesche WJ (1986). Role of *Streptococcus mutans* in human dental decay. Microbiol. Rev..

[32] Legenova K, Bujdakova H (2015). The role of *Streptococcus mutans* in the oral biofilm. Epidemiol. Mikrobiol. Imunol..

[33] Gross EL (2010). Bacterial 16S sequence analysis of severe caries in young permanent teeth. J. Clin. Microbiol..

[34] Nicolas GG, Lavoie MC (2011). *Streptococcus mutans* and oral streptococci in dental plaque. Can. J. Microbiol..

[35] Balakrishnan M, Simmonds RS, Tagg JR (2000). Dental caries is a preventable infectious disease. Aust. Dent. J..

[36] Bowen WH (2013). Rodent model in caries research. Odontology.

[37] Palmer CA (2010). Diet and caries-associated bacteria in severe early childhood caries. J. Dent. Res..

[38] Lin Y, Chen J, Zhou X, Li Y (2021). Inhibition of *Streptococcus mutans* biofilm formation by strategies targeting the metabolism of exopolysaccharides. Crit. Rev. Microbiol..

[39] Klein MI, Hwang G, Santos PHS, Campanella OH, Koo H (2015). *Streptococcus mutans*-derived extracellular matrix in cariogenic oral biofilms. Front. Cell. Infect. Microbiol..

[40] Pleszczynska M, Wiater A, Janczarek M, Szczodrak J (2015). (1->3)-α-D-glucan hydrolases in dental biofilm prevention and control: a review. Int. J. Biol. Macromol..

[41] Poulin MB, Kuperman LL (2021). Regulation of biofilm exopolysaccharide production by cyclic di-guanosine monophosphate. Front. Microbiol..

[42] Bowen WH, Burne RA, Wu H, Koo H (2018). Oral biofilms: pathogens, matrix and polymicrobial interactions in microenvironments. Trends Microbiol..

[43] Alves LA (2016). CovR regulates *Streptococcus mutans* susceptibility to complement immunity and survival in blood. Infect. Immun..

[44] Goodman SD (2011). Biofilms can be dispersed by focusing the immune system on a common family of bacterial nucleoid-associated proteins. Mucosal Immunol..

[45] Xiao J (2012). The exopolysaccharide matrix modulates the interaction between 3D architecture and virulence of a mixed-species oral biofilm. PLoS Pathog..

[46] Guo L, McLean JS, Lux R, He X, Shi W (2015). The well-coordinated linkage between acidogenicity and aciduricity via insoluble glucans on the surface of *Streptococcus mutans*. Sci. Rep..

[47] Banas JA (2004). Virulence properties of *Streptococcus mutans*. Front. Biosci. Landmark.

[48] Koo H, Allan RN, Howlin RP, Stoodley P, Hall-Stoodley L (2017). Targeting microbial biofilms: current and prospective therapeutic strategies. Nat. Rev. Microbiol..

[49] Matsumi Y (2015). Contribution of glucan-binding protein A to firm and stable biofilm formation by *Streptococcus mutans*. Mol. Oral Microbiol..

[50] Abranches, J. et al. Biology of oral streptococci. *Microbiol. Spectr*. **6**, 10.1128/microbiolspec.GPP3-0042-2018 (2018). 10.1128/microbiolspec.gpp3-0042-2018 PMC6287261 30338752

[51] Xu X, Zhou XD, Wu CD (2011). The tea catechin epigallocatechin gallate suppresses cariogenic virulence factors of *Streptococcus mutans*. Antimicrob. Agents Chemother..

[52] Ma Q (2022). Acetylation of lactate dehydrogenase negatively regulates the acidogenicity of *Streptococcus mutans*. mBio.

[53] Cotter PD, Hill C (2003). Surviving the acid test: responses of gram-positive bacteria to low pH. Microbiol. Mol. Biol. Rev..

[54] Liu Y-L, Nascimento M, Burne RA (2012). Progress toward understanding the contribution of alkali generation in dental biofilms to inhibition of dental caries. Int. J. Oral Sci..

[55] Li YH, Tian XL (2012). Quorum sensing and bacterial social interactions in biofilms. Sensors.

[56] Matsumoto-Nakano M (2018). Role of *Streptococcus mutans* surface proteins for biofilm formation. Jpn. Dent. Sci. Rev..

[57] Lei L (2015). Modulation of biofilm exopolysaccharides by the *Streptococcus mutans vicX* gene. Front. Microbiol..

[58] Sadeghinejad L (2017). Mechanistic, genomic and proteomic study on the effects of BisGMA-derived biodegradation product on cariogenic bacteria. Dent. Mater..

[59] Woelber JP, Al-Ahmad A, Alt KW (2022). On the pathogenicity of the oral biofilm: a critical review from a biological, evolutionary, and nutritional point of view. Nutrients.

[60] Dashiff A, Kadouri DE (2011). Predation of oral pathogens by *Bdellovibrio bacteriovorus* 109J. Mol. Oral Microbiol..

[61] Van Essche M (2011). Killing of anaerobic pathogens by predatory bacteria. Mol. Oral Microbiol..

[62] Zarco MF, Vess TJ, Ginsburg GS (2012). The oral microbiome in health and disease and the potential impact on personalized dental medicine. Oral Dis..

[63] Mercenier A, Pavan S, Pot B (2003). Probiotics as biotherapeutic agents: present knowledge and future prospects. Curr. Pharm. Des..

[64] Hill C (2014). The international scientific association for probiotics and prebiotics consensus statement on the scope and appropriate use of the term probiotic. Nat. Rev. Gastroenterol. Hepatol..

[65] Saiz P, Taveira N, Alves R (2021). Probiotics in oral health and disease: a systematic review. Appl. Sci..

[66] Simark-Mattsson C (2007). *Lactobacillus*-mediated interference of mutans streptococci in caries-free vs. caries-active subjects. Eur. J. Oral Sci..

[67] Inchingolo AD (2022). Oralbiotica/oralbiotics: the impact of oral microbiota on dental health and demineralization: a systematic review of the literature. Children.

[68] Teughels W, Van Essche M, Sliepen I, Quirynen M (2008). Probiotics and oral healthcare. Periodontology.

[69] Talarico TL, Casas IA, Chung TC, Dobrogosz WJ (1988). Production and isolation of reuterin, a growth inhibitor produced by *Lactobacillus reuteri*. Antimicrob. Agents Chemother..

[70] Gänzle MG, Höltzel A, Walter J, Jung G, Hammes WP (2000). Characterization of reutericyclin produced by *Lactobacillus reuteri* LTH2584. Appl. Environ. Microbiol..

[71] Caglar E (2005). Effect of yogurt with *Bifidobacterium* DN-173 010 on salivary mutans streptococci and lactobacilli in young adults. Acta Odontol. Scand..

[72] Darbandi A (2022). Bacteriocins: properties and potential use as antimicrobials. J. Clin. Lab. Anal..

[73] Rogers LA (1928). The inhibiting effect of *Streptococcus lactis* on *Lactobacillus bulgaricus*. J. Bacteriol..

[74] Heng BC (2007). Reluctance of medical professionals in adopting natural-cycle and minimal ovarian stimulation protocols in human clinical assisted reproduction. Reprod. Biomed. Online.

[75] Wang Y, Qin Y, Zhang Y, Wu R, Li P (2019). Antibacterial mechanism of plantaricin LPL-1, a novel class IIa bacteriocin against Listeria monocytogenes. Food Control.

[76] Surachat K, Sangket U, Deachamag P, Chotigeat W (2017). In silico analysis of protein toxin and bacteriocins from *Lactobacillus paracasei* SD1 genome and available online databases. PLoS One.

[77] Nagao J (2006). Lantibiotics: insight and foresight for new paradigm. J. Biosci. Bioeng..

[78] Yang S-C, Lin C-H, Sung CT, Fang J-Y (2014). Antibacterial activities of bacteriocins: application in foods and pharmaceuticals. Front. Microbiol..

[79] Jin X, An S, Kightlinger W, Zhou J, Hong SH (2021). Engineering *Escherichia coli* to produce and secrete colicins for rapid and selective biofilm cell killing. AIChE J..

[80] Dobson A, Cotter PD, Ross RP, Hill C (2012). Bacteriocin production: a probiotic trait?. Appl. Environ. Microbiol..

[81] Radaic A (2020). Modulation of pathogenic oral biofilms towards health with nisin probiotic. J. Oral. Microbiol..

[82] Conrads G, Westenberger J, Luerkens M, Abdelbary MMH (2019). Isolation and bacteriocin-related typing of *Streptococcus dentisani*. Front. Cell Infect. Microbiol..

[83] Jaffar N, Ishikawa Y, Mizuno K, Okinaga T, Maeda T (2016). Mature biofilm degradation by potential probiotics: *Aggregatibacter actinomycetemcomitans* versus *Lactobacillus* spp. PLoS One.

[84] Walker GV (2016). Salivaricin E and abundant dextranase activity may contribute to the anti-cariogenic potential of the probiotic candidate *Streptococcus salivarius* JH. Microbiology.

[85] Huang X (2016). A highly arginolytic *Streptococcus* species that potently antagonizes *Streptococcus mutans*. Appl. Environ. Microbiol..

[86] Di Pierro F, Zanvit A, Nobili P, Risso P, Fornaini C (2015). Cariogram outcome after 90 days of oral treatment with *Streptococcus salivarius* M18 in children at high risk for dental caries: results of a randomized, controlled study. Clin. Cosmet. Investig. Dent..

[87] Satpute SK (2016). Biosurfactant/s from lactobacilli species: properties, challenges and potential biomedical applications. J. Basic Microbiol..

[88] Sharma D, Singh Saharan B (2014). Simultaneous production of biosurfactants and bacteriocins by probiotic *Lactobacillus casei* MRTL3. Int. J. Microbiol..

[89] Rodrigues LR, Teixeira JA, Oliveira R (2006). Low-cost fermentative medium for biosurfactant production by probiotic bacteria. Biochem. Eng. J..

[90] Saravanakumari P, Mani K (2010). Structural characterization of a novel xylolipid biosurfactant from *Lactococcus lactis* and analysis of antibacterial activity against multi-drug resistant pathogens. Bioresour. Technol..

[91] Thavasi R, Jayalakshmi S, Banat IM (2011). Effect of biosurfactant and fertilizer on biodegradation of crude oil by marine isolates of *Bacillus megaterium*, *Corynebacterium kutscheri* and *Pseudomonas aeruginosa*. Bioresour. Technol..

[92] Ciandrini E (2016). Characterization of biosurfactants produced by *Lactobacillus* spp. and their activity against oral streptococci biofilm. Appl. Microbiol. Biotechnol..

[93] Tahmourespour A, Salehi R, Kasra Kermanshahi R (2011). *Lactobacillus acidophilus*-derived biosurfactant effect on *gtfB* and *gtfC* expression level in *Streptococcus mutans* biofilm cells. Braz. J. Microbiol..

[94] Tan Y, Leonhard M, Moser D, Schneider-Stickler B (2017). Inhibition activity of *Lactobacilli* supernatant against fungal-bacterial multispecies biofilms on silicone. Microb. Pathog..

[95] Gudina EJ, Teixeira JA, Rodrigues LR (2010). Isolation and functional characterization of a biosurfactant produced by *Lactobacillus paracasei*. Colloids Surf. B Biointerfaces.

[96] Özcelik S, Kuley E, Özogul F (2016). Formation of lactic, acetic, succinic, propionic, formic and butyric acid by lactic acid bacteria. LWT Food Sci. Technol..

[97] Lin X, Chen X, Chen Y, Jiang W, Chen H (2015). The effect of five probiotic lactobacilli strains on the growth and biofilm formation of *Streptococcus mutans*. Oral Dis..

[98] Bustamante M, Oomah BD, Mosi-Roa Y, Rubilar M, Burgos-Diaz C (2020). Probiotics as an adjunct therapy for the treatment of halitosis, dental caries and periodontitis.. Probiotics Antimicrob. Proteins.

[99] Redanz S (2018). Live and let die: hydrogen peroxide production by the commensal flora and its role in maintaining a symbiotic microbiome. Mol. Oral Microbiol..

[100] Herrero ER (2016). Antimicrobial effects of commensal oral species are regulated by environmental factors. J. Dent..

[101] El Oirdi S (2021). Isolation and identification of *Lactobacillus plantarum* 4F, a strain with high antifungal activity, fungicidal effect, and biopreservation properties of food. J. Food Process. Preserv..

[102] Lai W-K (2021). Developing lactic acid bacteria as an oral healthy food. Life.

[103] Barzegari A (2020). The battle of probiotics and their derivatives against biofilms. Infect. Drug Resist..

[104] Wasfi R, Abd El-Rahman OA, Zafer MM, Ashour HM (2018). Probiotic *Lactobacillus* sp. inhibit growth, biofilm formation and gene expression of caries-inducing *Streptococcus* mutans. J. Cell. Mol. Med..

[105] Matsubara VH, Wang Y, Bandara HMHN, Mayer MPA, Samaranayake LP (2016). Probiotic lactobacilli inhibit early stages of *Candida albicans* biofilm development by reducing their growth, cell adhesion, and filamentation. Appl. Microbiol. Biotechnol..

[106] James KM, MacDonald KW, Chanyi RM, Cadieux PA, Burton JP (2016). Inhibition of *Candida albicans* biofilm formation and modulation of gene expression by probiotic cells and supernatant. J. Med. Microbiol..

[107] Cortes-Acha B (2019). Development and viability of biofilms grown on experimental abutments mimicking dental implants: an in vivo model. Med. Oral Patol. Oral. Cir. Bucal.

[108] Jung H-Y (2022). Collagen peptide in a combinatorial treatment with *Lactobacillus rhamnosus* inhibits the cariogenic properties of *Streptococcus mutans*: an in vitro study. Int. J. Mol. Sci..

[109] Lin T-H, Lin C-H, Pan T-M (2018). The implication of probiotics in the prevention of dental caries. Appl. Microbiol. Biotechnol..

[110] Singh TP, Kaur G, Kapila S, Malik RK (2017). Antagonistic activity of *Lactobacillus reuteri* strains on the adhesion characteristics of selected pathogens. Front. Microbiol..

[111] Burton JP (2013). Influence of the probiotic *Streptococcus salivarius* strain M18 on indices of dental health in children: a randomized double-blind, placebo-controlled trial. J. Med. Microbiol..

[112] Ha Kim J, Jang HJ, Lee N-K, Paik H-D (2022). Antibacterial and antibiofilm effect of cell-free supernatant of *Lactobacillus brevis* KCCM 202399 isolated from korean fermented food against *Streptococcus mutans* KCTC 5458. J. Microbiol. Biotechnol..

[113] Haukioja A, Loimaranta V, Tenovuo J (2008). Probiotic bacteria affect the composition of salivary pellicle and streptococcal adhesion in vitro. Oral Microbiol. Immunol..

[114] Tenovuo J (1998). Antimicrobial function of human saliva-how important is it for oral health?. Acta Odontol. Scand..

[115] Boris S, Suárez JE, Barbés C (1997). Characterization of the aggregation promoting factor from *Lactobacillus gasseri*, a vaginal isolate. J. Appl. Microbiol..

[116] Lang C (2010). Specific *Lactobacillus*/mutans Streptococcus co-aggregation. J. Dent. Res.

[117] Sliepen I (2009). Microbial interactions influence inflammatory host cell responses. J. Dent. Res..

[118] Wattanarat O (2021). Significant elevation of salivary human neutrophil peptides 1-3 levels by probiotic milk in preschool children with severe early childhood caries: a randomized controlled trial. Clin. Oral Investig..

[119] Pahumunto N, Sophatha B, Piwat S, Teanpaisan R (2019). Increasing salivary IgA and reducing *Streptococcus mutans* by probiotic *Lactobacillus paracasei* SD1: a double-blind, randomized, controlled study. J. Dent. Sci..

[120] Balzaretti S (2017). A novel rhamnose-rich hetero-exopolysaccharide isolated from *Lactobacillus paracasei* DG activates THP-1 human monocytic cells. Appl. Environ. Microbiol..

[121] Amargianitakis M, Antoniadou M, Rahiotis C, Varzakas T (2021). Probiotics, prebiotics, synbiotics and dental caries. new perspectives, suggestions, and patient coaching approach for a cavity-free mouth. Appl. Sci..

[122] Nadelman P, Magno MB, Masterson D, da Cruz AG, Maia LC (2018). Are dairy products containing probiotics beneficial for oral health? a systematic review and meta-analysis. Clin. Oral Investig..

[123] Gedalia I (1991). Enamel softening with Coca-Cola and rehardening with milk or saliva. Am. J. Dent..

[124] Kashket S, Yaskell T (1997). Effectiveness of calcium lactate added to food in reducing intraoral demineralization of enamel. Caries Res..

[125] Schüpbach P, Neeser JR, Golliard M, Rouvet M, Guggenheim B (1996). Incorporation of caseinoglycomacropeptide and caseinophosphopeptide into the salivary pellicle inhibits adherence of mutans streptococci. J. Dent. Res..

[126] Swarna SK, Nivedhitha MS (2020). Probiotics in prevention of dental caries—a literature review. Biosci. Biotechnol. Res. Commun..

[127] de Alvarenga JA (2021). Probiotic effects of *lactobacillus paracasei* 28.4 to inhibit *Streptococcus mutans* in a gellan-based formulation.. Probiotics Antimicrob. Proteins.

[128] Yelin I (2019). Genomic and epidemiological evidence of bacterial transmission from probiotic capsule to blood in ICU patients. Nat. Med..

[129] Gruner D, Paris S, Schwendicke F (2016). Probiotics for managing caries and periodontitis: systematic review and meta-analysis. J. Dent..

[130] Corby PM (2005). Microbial risk indicators of early childhood caries. J. Clin. Microbiol..

[131] Wen ZT, Huang X, Ellepola K, Liao S, Li Y (2022). Lactobacilli and human dental caries: more than mechanical retention. Microbiology.

[132] Henne K, Rheinberg A, Melzer-Krick B, Conrads G (2015). Aciduric microbial taxa including *Scardovia wiggsiae* and *Bifidobacterium* spp. in caries and caries free subjects. Anaerobe.

[133] Caufield PW, Schön CN, Saraithong P, Li Y, Argimón S (2015). Oral lactobacilli and dental caries: a model for niche adaptation in humans. J. Dent. Res..

[134] Newhouse MT, Dolovich M (1986). Spacer devices for asthma. J. Pediatr..

[135] Gibson GR, Roberfroid MB (1995). Dietary modulation of the human colonic microbiota: introducing the concept of prebiotics. J. Nutr..

[136] Gibson GR (2017). Expert consensus document: the international scientific association for probiotics and prebiotics (ISAPP) consensus statement on the definition and scope of prebiotics. Nat. Rev. Gastroenterol. Hepatol..

[137] Guerrero-Wyss M, Durán Agüero S, Angarita Dávila L (2018). D-tagatose is a promising sweetener to control glycaemia: a new functional food. Biomed. Res. Int..

[138] Mayumi S (2021). Potential of prebiotic D-tagatose for prevention of oral disease. Front. Cell Infect. Microbiol..

[139] Nagamine Y (2020). D-tagatose effectively reduces the number of *Streptococcus mutans* and oral bacteria in healthy adult subjects: a chewing gum pilot study and randomized clinical trial. Acta Med. Okayama.

[140] Kojima Y, Ohshima T, Seneviratne CJ, Maeda N (2016). Combining prebiotics and probiotics to develop novel synbiotics that suppress oral pathogens. J. Oral Biosci..

[141] Söderling E, Pienihäkkinen K (2020). Effects of xylitol and erythritol consumption on mutans streptococci and the oral microbiota: a systematic review. Acta Odontol. Scand..

[142] Gibson GR, Probert HM, Loo JV, Rastall RA, Roberfroid MB (2004). Dietary modulation of the human colonic microbiota: updating the concept of prebiotics. Nutr. Res. Rev..

[143] Roberfroid M (2010). Prebiotic effects: metabolic and health benefits. Br. J. Nutr..

[144] Cocco F (2017). The caries preventive effect of 1-year use of low-dose xylitol chewing gum. a randomized placebo-controlled clinical trial in high-caries-risk adults. Clin. Oral Investig..

[145] Söderling E, Alaräisänen L, Scheinin A, Mäkinen KK (1987). Effect of xylitol and sorbitol on polysaccharide production by and adhesive properties of *Streptococcus mutans*. Caries Res..

[146] Watthanasaen S (2017). Xylitol-containing chewing gum for caries prevention in students with disabilities: a randomised trial. Oral Health Prev. Dent..

[147] Gauthier L, Vadeboncoeur C, Mayrand D (1984). Loss of sensitivity to xylitol by *Streptococcus mutans* LG-1. Caries Res..

[148] Falony G (2016). Long-term effect of erythritol on dental caries development during childhood: a posttreatment survival analysis. Caries Res..

[149] Thabuis C (2013). Effects of maltitol and xylitol chewing-gums on parameters involved in dental caries development. Eur. J. Paediatr. Dent..

[150] Salli K, Söderling E, Hirvonen J, Gürsoy UK, Ouwehand AC (2020). Influence of 2′-fucosyllactose and galacto-oligosaccharides on the growth and adhesion of *Streptococcus mutans*. Br. J. Nutr..

[151] Sharon N (2006). Carbohydrates as future anti-adhesion drugs for infectious diseases. Biochim. Biophys. Acta.

[152] Oku T, Nakamura S (2007). Threshold for transitory diarrhea induced by ingestion of xylitol and lactitol in young male and female adults. J. Nutr. Sci. Vitaminol..

[153] Koopman JE (2015). Stability and resilience of oral microcosms toward acidification and *Candida* outgrowth by arginine supplementation. Microb. Ecol..

[154] Bacali C (2022). Oral microbiome: getting to know and befriend neighbors, a biological approach. Biomedicines.

[155] Zheng X (2017). Ecological effect of arginine on oral microbiota. Sci. Rep..

[156] He J (2016). L-arginine modifies the exopolysaccharide matrix and thwarts *Streptococcus mutans* outgrowth within mixed-species oral biofilms. J. Bacteriol..

[157] Koopman JE (2017). Changes in the oral ecosystem induced by the use of 8% arginine toothpaste. Arch. Oral Biol..

[158] Yin W (2013). The anti-caries efficacy of a dentifrice containing 1.5% arginine and 1450 ppm fluoride as sodium monofluorophosphate assessed using quantitative light-induced fluorescence (QLF). J. Dent..

[159] Bijle MNA, Ekambaram M, Lo EC, Yiu CKY (2018). The combined enamel remineralization potential of arginine and fluoride toothpaste. J. Dent..

[160] Carda-Diéguez M, Moazzez R, Mira A (2022). Functional changes in the oral microbiome after use of fluoride and arginine containing dentifrices: a metagenomic and metatranscriptomic study. Microbiome.

[161] Cheng X (2020). Magnesium-dependent promotion of H_2_O_2_ production increases ecological competitiveness of oral commensal streptococci. J. Dent. Res..

[162] Burne RA, Marquis RE (2000). Alkali production by oral bacteria and protection against dental caries. FEMS Microbiol. Lett..

[163] Zaura E, Twetman S (2019). Critical appraisal of oral pre- and probiotics for caries prevention and care. Caries Res..

[164] Sánchez GA, Miozza VA, Delgado A, Busch L (2014). Total salivary nitrates and nitrites in oral health and periodontal disease. Nitric Oxide.

[165] Doel JJ (2004). Protective effect of salivary nitrate and microbial nitrate reductase activity against caries. Eur. J. Oral Sci..

[166] Green SJ (1995). Nitric oxide in mucosal immunity. Nat. Med..

[167] Allaker RP, Silva Mendez LS, Hardie JM, Benjamin N (2001). Antimicrobial effect of acidified nitrite on periodontal bacteria. Oral Microbiol. Immunol..

[168] Rosier BT, Buetas E, Moya-Gonzalvez EM, Artacho A, Mira A (2020). Nitrate as a potential prebiotic for the oral microbiome. Sci. Rep..

[169] Li H (2007). Salivary nitrate—an ecological factor in reducing oral acidity. Oral Microbiol. Immunol..

[170] Jockel-Schneider Y (2016). Stimulation of the nitrate-nitrite-NO-metabolism by repeated lettuce juice consumption decreases gingival inflammation in periodontal recall patients: a randomized, double-blinded, placebo-controlled clinical trial. J. Clin. Periodontol..

[171] Gee LC, Ahluwalia A (2016). Dietary nitrate lowers blood pressure: epidemiological, pre-clinical experimental and clinical trial evidence. Curr. Hypertens. Rep..

[172] Vanhatalo A (2018). Nitrate-responsive oral microbiome modulates nitric oxide homeostasis and blood pressure in humans. Free Radic. Biol. Med..

[173] Velmurugan S (2015). Dietary nitrate improves vascular function in patients with hypercholesterolemia: a randomized, double-blind, placebo-controlled study. Am. J. Clin. Nutr..

[174] Markowiak P, Śliżewska K (2017). Effects of probiotics, prebiotics, and synbiotics on human health. Nutrients.

[175] Swanson KS (2020). The international scientific association for probiotics and prebiotics (ISAPP) consensus statement on the definition and scope of synbiotics. Nat. Rev. Gastroenterol. Hepatol..

[176] Nunpan S, Suwannachart C, Wayakanon K (2019). Effect of prebiotics-enhanced probiotics on the growth of *Streptococcus mutans*. Int. J. Microbiol..

[177] Tester R, Al-Ghazzewi F (2011). A preliminary study of the synbiotic effects of konjac glucomannan hydrolysates (GMH) and lactobacilli on the growth of the oral bacterium *Streptococcus mutans*. Nutr. Food Sci..

[178] Bijle MN, Neelakantan P, Ekambaram M, Lo ECM, Yiu CKY (2020). Effect of a novel synbiotic on *Streptococcus mutans*. Sci. Rep..

[179] Salminen S (2021). The international scientific association of probiotics and prebiotics (ISAPP) consensus statement on the definition and scope of postbiotics. Nat. Rev. Gastroenterol. Hepatol..

[180] Barros CP (2020). Paraprobiotics and postbiotics: concepts and potential applications in dairy products. Curr. Opin. Food Sci..

[181] Moradi M (2020). Postbiotics produced by lactic acid bacteria: the next frontier in food safety. Compr. Rev. Food Sci. Food Saf..

[182] Holz C (2013). *Lactobacillus paracasei* DSMZ16671 reduces mutans Streptococci: a short-term pilot study.. Probiotics Antimicrob. Proteins.

[183] Moradi M, Molaei R, Guimarães JT (2021). A review on preparation and chemical analysis of postbiotics from lactic acid bacteria. Enzym. Microb. Technol..

[184] el-Nezami H, Kankaanpää P, Salminen S, Ahokas J (1998). Physicochemical alterations enhance the ability of dairy strains of lactic acid bacteria to remove aflatoxin from contaminated media. J. Food Prot..

[185] Schwendicke F, Horb K, Kneist S, Dörfer C, Paris S (2014). Effects of heat-inactivated *Bifidobacterium* BB12 on cariogenicity of *Streptococcus mutans* in vitro. Arch. Oral Biol..

[186] Tareb R, Bernardeau M, Gueguen M, Vernoux J-P (2013). In vitro characterization of aggregation and adhesion properties of viable and heat-killed forms of two probiotic *Lactobacillus* strains and interaction with foodborne zoonotic bacteria, especially *Campylobacter jejuni*. J. Med. Microbiol..

[187] Pahumunto N (2018). Reducing mutans streptococci and caries development by *Lactobacillus paracasei* SD1 in preschool children: a randomized placebo-controlled trial. Acta Odontol. Scand..

[188] Ritthagol W, Saetang C, Teanpaisan R (2014). Effect of probiotics containing *Lactobacillus paracasei* SD1 on salivary mutans streptococci and lactobacilli in orthodontic cleft patients: a double-blinded, randomized, placebo-controlled study. Cleft Palate Craniofac. J..

[189] Nozari A, Motamedifar M, Seifi N, Hatamizargaran Z, Ranjbar MA (2015). The effect of Iranian customary used probiotic yogurt on the children’s salivary cariogenic microflora. J. Dent..

[190] Pinto GS, Cenci MS, Azevedo MS, Epifanio M, Jones MH (2014). Effect of yogurt containing *Bifidobacterium animalis* subsp. *lactis* DN-173010 probiotic on dental plaque and saliva in orthodontic patients. Caries Res..

[191] Zare Javid A (2020). Effects of the consumption of probiotic yogurt containing *Bifidobacterium lactis* Bb12 on the levels of *Streptococcus mutans* and lactobacilli in saliva of students with initial stages of dental caries: a double-blind randomized controlled trial. Caries Res..

[192] Miyazima T, Ishikawa K, Mayer M, Saad S, Nakamae A (2017). Cheese supplemented with probiotics reduced the *Candida* levels in denture wearers—RCT. Oral Dis..

[193] Ahola AJ (2002). Short-term consumption of probiotic-containing cheese and its effect on dental caries risk factors. Arch. Oral Biol..

[194] Mortazavi S, Akhlaghi N (2012). Salivary *Streptococcus mutans* and *Lactobacilli* levels following probiotic cheese consumption in adults: a double blind randomized clinical trial*. J. Res. Med. Sci..

[195] Ashwin D (2015). Effect of probiotic containing ice-cream on salivary mutans streptococci (SMS) levels in children of 6-12 years of age: a randomized controlled double blind study with six-months follow up. J. Clin. Diagn. Res..

[196] Hasslof P, West CE, Videhult FK, Brandelius C, Stecksen-Blicks C (2013). Early intervention with probiotic *Lactobacillus paracasei* F19 has no long-term effect on caries experience. Caries Res..

[197] Taipale T, Pienihakkinen K, Salminen S, Jokela J, Soderling E (2012). *Bifidobacterium animalis* subsp. *lactis* BB-12 administration in early childhood: a randomized clinical trial of effects on oral colonization by mutans streptococci and the probiotic. Caries Res..

[198] Caglar E (2007). Effect of chewing gums containing xylitol or probiotic bacteria on salivary mutans streptococci and lactobacilli. Clin. Oral Investig..

[199] Srivastava S, Saha S, Kumari M, Mohd S (2016). Effect of probiotic curd on salivary pH and *Streptococcus mutans*: a double blind parallel randomized controlled trial. J. Clin. Diagn. Res..

[200] Jose JE, Padmanabhan S, Chitharanjan AB (2013). Systemic consumption of probiotic curd and use of probiotic toothpaste to reduce *Streptococcus mutans* in plaque around orthodontic brackets. Am. J. Orthod. Dentofac. Orthop..

[201] Pohjavuori S (2010). Effect of consumption of *Lactobacillus rhamnosus* GG and calcium, in carrot-pineapple juice on dental caries risk in children. Int. J. Probiotics Prebiotics.

[202] Zahradnik RT (2009). Preliminary assessment of safety and effectiveness in humans of ProBiora^3 TM^, a probiotic mouthwash. J. Appl. Microbiol..

